# Vertebrate Left-Right Asymmetry: What Can Nodal Cascade Gene Expression Patterns Tell Us?

**DOI:** 10.3390/jcdd5010001

**Published:** 2017-12-29

**Authors:** Axel Schweickert, Tim Ott, Sabrina Kurz, Melanie Tingler, Markus Maerker, Franziska Fuhl, Martin Blum

**Affiliations:** Institute of Zoology, University of Hohenheim, 70593 Stuttgart, Germany; axel.schweickert@uni-hohenheim.de (A.S.); tim.ott@uni-hohenheim.de (T.O.); sabrina.kurz@uni-hohenheim.de (S.K.); melanie.tingler@uni-hohenheim.de (M.T.); markus.maerker@uni-hohenheim.de (M.M.); franziska.fuhl@uni-hohenheim.de (F.F.)

**Keywords:** left-right asymmetry, symmetry breaking, cilia, Nodal, leftward flow, left-right organizer, Nodal cascade, heterotaxia, situs ambiguus, situs inversus

## Abstract

Laterality of inner organs is a wide-spread characteristic of vertebrates and beyond. It is ultimately controlled by the left-asymmetric activation of the Nodal signaling cascade in the lateral plate mesoderm of the neurula stage embryo, which results from a cilia-driven leftward flow of extracellular fluids at the left-right organizer. This scenario is widely accepted for laterality determination in wildtype specimens. Deviations from this norm come in different flavors. At the level of organ morphogenesis, laterality may be inverted (situs inversus) or non-concordant with respect to the main body axis (situs ambiguus or heterotaxia). At the level of Nodal cascade gene activation, expression may be inverted, bilaterally induced, or absent. In a given genetic situation, patterns may be randomized or predominantly lacking laterality (absence or bilateral activation). We propose that the distributions of patterns observed may be indicative of the underlying molecular defects, with randomizations being primarily caused by defects in the flow-generating ciliary set-up, and symmetrical patterns being the result of impaired flow sensing, on the left, the right, or both sides. This prediction, the reasoning of which is detailed in this review, pinpoints functions of genes whose role in laterality determination have remained obscure.

## 1. Introduction

The development of the left-right body axis in an otherwise bilaterally symmetrical organism has drawn the attention of anatomists and developmental biologists alike at all times [1,2,3]. Asymmetric positioning and morphogenesis of many organs (*situs solitus*), including heart, lung, gut, liver, and stomach, is relevant to humans, as deviations from the norm impact on health or are even incompatible with life. A mirror-imaged organ placement (*situs inversus*), however, mostly lacks clinical relevance, in stark contrast to the misplacement of single organs (*situs ambiguus* or heterotaxia) or duplications of sided arrangements (left or right isomerism), which often cause embryonic lethality [4]. The molecular analysis of left-right (LR) development was initiated by the identification of left-asymmetrically expressed genes during neurula stages of chick, mouse, and frog embryos [5,6,7]. The TGFβ-type morphogene Nodal, its secreted feedback inhibitor Lefty, and the homeobox transcription factor Pitx2 are activated in a sided manner in the left lateral plate mesoderm (LPM) and make up the so-called Nodal cascade. Once activated, Nodal signaling directly induces its own transcription, resulting in the fast spreading of *nodal* mRNA expression in the LPM [8]. In addition, Nodal activates *lefty* and *pitx2* transcription. *Nodal* expression vanishes after a very short time, due to the feedback inhibition by Lefty, whereas left-sided *pitx2* expression is maintained up to organ morphogenesis, which is initiated right after *Nodal* has disappeared from the LPM [9]. Several mutants affect this narrow timing, such as, for example, *dand5* in the mouse [10]. Loss-of-function experiments either targeting Nodal signaling or Pitx2 resulted in aberrant organ situs in all animal model systems analyzed, demonstrating the conserved requirement of the Nodal-Lefty-Pitx2 cassette for LR development [5,11]. The morphogen nodal, known to act as a long range signaling molecule, is further restricted by Lefty expression at the midline. Defective midline Lefty enables Nodal to diffuse to the right LPM and to activate a right-sided cascade, resulting in LR defects as well [12]. At an evolutionary scale, the Nodal cascade governs asymmetries in the entire deuterostome tree of life, from sea urchins to mammals [13,14].

**Figure 1:**
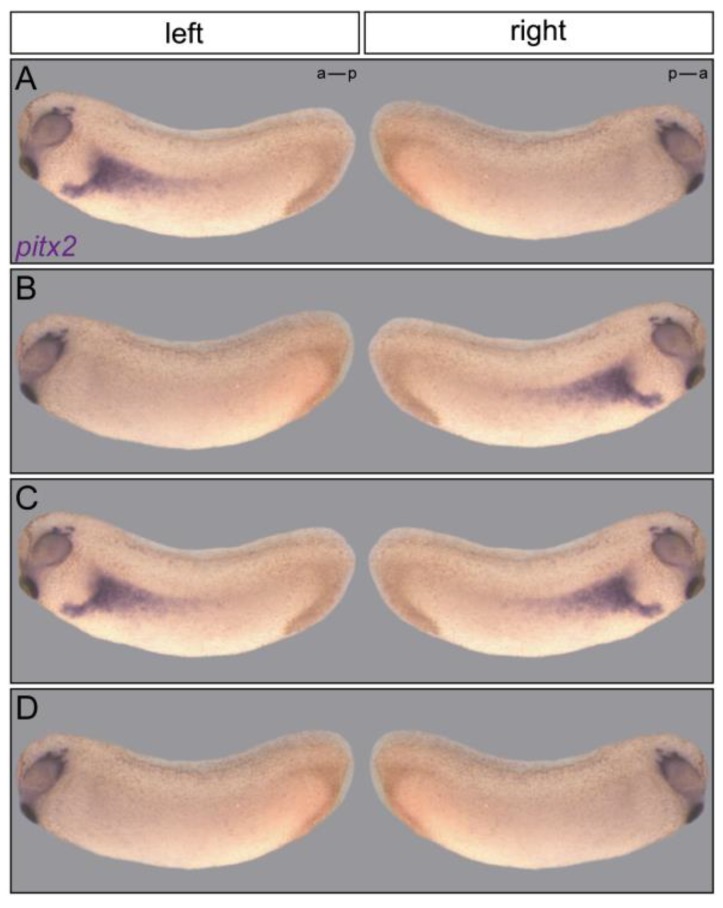
Expression patterns of Nodal cascade genes. Shown are *Pitx2* gene expression patterns in *Xenopus* tadpoles that can be wildtype (**A**), inverted (**B**), bilateral (**C**), or absent (**D**) on both sides, such as encountered in mutants and morphants of the dynein motor protein defective in *iv* mutant mice.

Besides evolutionary implications, the presence of the Nodal cascade at the center of events leading up to asymmetric organ placement and morphogenesis has enabled the analysis of processes upstream and downstream [15,16]. A relatively strong correlation between organ situs and Nodal cascade induction has been observed in many mouse mutants, best exemplified by the classical LR mutant *inversus viscerum,* or *iv* [17,18,19]. Nodal cascade induction is completely randomized in *iv* mutant embryos, with equal 25% frequencies of wildtype, absent, bilateral and right-sided expression patterns each (Figure 1). The organs of homozygous *iv* mutants are either wt (*situs solitus*; SS), inverted (*situs inversus*; SI), or mirror images of the left or right body half (left or right isomerism) [17,18,19], although deviations from this predicted distribution were recorded as well [20]. *iv* specimens with isomerisms die *in utero*, while both SS and SI animals are born and vital. This correlation is less clear in other mutants, especially when aberrant organ situs is only encountered in a minority of cases. Compensation of germ line mutations is only beginning to be studied, let alone to be understood, but may contribute in cases when Nodal cascade expression patterns and organ situs correlate less well [21,22]. Lower vertebrates, i.e., fish and frog, for the most parts lack isomerisms at the organ level. Absent or bilateral expression of Nodal cascade genes therefore result in the same set of phenotypes, *situs solitus*, *situs inversus,* and *situs ambiguus* (heterotaxia). This observation suggests that gastrointestinal and heart primordia of lower vertebrates are able to undergo an intrinsic chiral morphogenesis, leading to asymmetric organ placement even when positional information by the Nodal cascade is lacking or present on both sides. Up to now, these differences of asymmetric organ morphogenesis between species have not been systematically addressed. When considering all cases, however, it seems safe to hypothesize that Nodal cascade gene expression patterns in the LPM are indicative of organ situs development. In the context of the present discussion, we shall therefore exclusively consider Nodal cascade patterns as indicators of upstream mechanisms. In the following, we will take what may be considered a somewhat unusual approach of a more theoretical look at concepts of flow generation, sensing and signal transfer. In some cases, this will result in over-simplifications that neglect the complexity of phenotypes encountered in mutants and morphants, for which we apologize, as we do to all colleagues whose work we could not cite due to space restrictions.

## 2. Cilia-Dependent Symmetry Breaking

**Figure 2:**
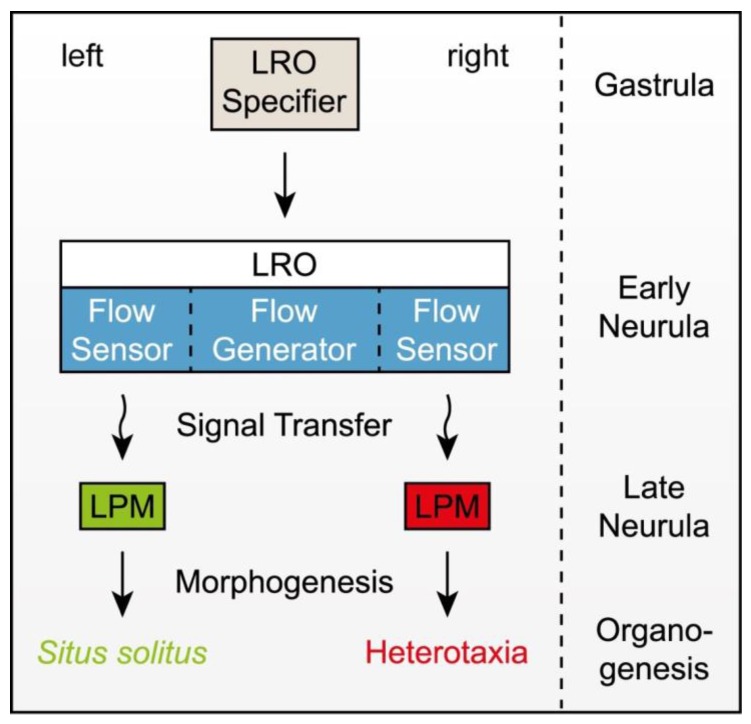
Schematic depiction of left-right (LR) axis specification in vertebrate embryos that use a ciliated left-right organizer (LRO) to break the bilateral symmetry of the early embryo. For a detailed discussion, see main text.

While the mechanisms of asymmetric organ morphogenesis downstream of the Nodal cascade are only slowly beginning to be unraveled (cf. [16] for a recent review), upstream events have received much attention in the various model organisms and a great deal of knowledge has accumulated in recent years [15]. In a sense, the *iv* mutant again has paved the way: the identification of its target gene as an axonemal dynein motor has introduced cilia as a central player, with a multitude of ciliary mutants being involved in LR development and a great many of ciliopathies displaying LR defects as one of their characteristics [18]. In short, the situation as of today presents as follows (Figure 2): in the perfectly bilateral symmetrical neurula embryo, the ciliated left-right organizer (LRO) forms at the midline. Precursors arise during gastrulation and are patterned by the embryonic organizer (Figure 2). The LRO creates an extracellular fluid flow from the right to the left side. This leftward flow may or may not transport cargo, which should originate from within the LRO. The LRO harbors flow sensors at the margins on both sides, which react to flow and/or to the cargo when it gets delivered to the sensor. The sensor generates a signal that gets transmitted to the LPM, where it induces the Nodal cascade. Any disturbance of this sequence of events, which together rules symmetry breaking in the embryo, inevitably results in alterations of LPM Nodal cascade expression. However, the type of alteration observed does not necessarily pinpoint the underlying defects in symmetry breaking. For example, it is not easily reconciled that in a mutant such as *iv,* which renders cilia immotile one quarter of specimens develop without any LR defects, although left LPM Nodal in *iv* is not induced by the exactly same mechanism as in wildtype embryos [23,24]. Such cases have in the past led to the proposal that it is utterly impossible that cilia take center stage in symmetry breaking [25,26,27], and they continue to plague the field, as honestly nobody knows how left-asymmetric Nodal is induced in the absence of ciliary motility or in any of the other mutants that eventually show *situs solitus* in at least a proportion of homozygous specimens.

We propose that it is profitable to think about disturbances of distinct steps in the chain of events that account for symmetry breaking, and to predict what kind of alterations of Nodal cascade gene expression patterns should result. Some mutants will fall in one or another category, in some other cases combinations of defects may cause a specific pattern. 

### 2.1. LRO Specifier

To start the discussion, we first like to consider global LRO specifiers, i.e., mutants in which an LRO does not form at all. So far, LRO precursors have only been identified in fish and frog with the dorsal forerunner cells and the superficial mesoderm, respectively [28,29]. From the timing and geometry of embryonic development, the generation of these LRO specifiers must be intimately linked to the function of the primary embryonic (or Spemann) organizer. LROs inevitably localize to the posterior end of the notochord, which develops from the organizer [30]. Interestingly, mechanical ablations of frog and fish LROs have not impacted on embryonic development beyond laterality determination, demonstrating that symmetry breaking is a distinct event [31,32,33]. Quite obviously, if no LRO is specified, then the Nodal cascade should not be induced, i.e., LPM gene expression should be absent. Several mutants are known in which LRO formation is distorted, but only one in which this ciliated epithelium does not form at all: *brachyury*. To no surprise, the LPM Nodal cascade is not induced, and this is true for mouse mutants [34,35,36] as well as frog morphants (SK and MB, unpublished. In zebrafish, although the LRO is missing (with flow generator and sensor being absent, see below), LPM *nodal* is induced (REF), which remains a mystery. Another prominent gene that affects LRO morphogenesis is *noto* [32,37]. In contrast to *brachyury*, LRO remnants form, for example, in zebrafish, where Kupffer’s vesicle is present but much reduced in size [28]. In this case, the Nodal cascade is induced in a bilateral manner, i.e., both in the left and right LPM. Interestingly, LRO specifier genes are generally required for notochord formation, in agreement with the notion that LRO and notochord morphogenesis are intimately linked [30]. The exact pathway how these genes set up this unique tissue during gastrulation has not been elucidated as yet, but mutants that abrogate LRO formation should always result in the absence of Nodal cascade gene induction.

### 2.2. Flow Generator

We next consider flow generators, i.e., cells that harbor motile cilia. These are found at the center of the LRO and may be present or absent and harbor cilia or not. If cilia are present, then they may be motile or immotile, of correct length, morphology, and posterior polarization, or not. As a result, leftward flow may proceed with normal speed and directionality or deviate from the norm. All of the deviations should result in altered LPM expression patterns. But what is to be expected in every single case? Mispolarized cilia should produce fluid flows that are directed to other dimensions than left-only. In such settings, the Nodal cascade might be induced on the right side. No case is known in which all cilia polarize in the wrong (anterior) direction and therefore invert the cascade, but several genes are known that result in a proportion of anteriorly polarized cilia. In such cases, which comprise some of the planar cell polarity genes, such as *vangl1/2* and *wnt11,* as well as the ion pump ATP4 [38,39,40,41], bilateral induction of the Nodal cascade has been reported (Table 1). Variations in cilia lengths cause altered flow dynamics and LR defects, as recently shown in zebrafish [42]. Deviations in speed of leftward flow have not systematically been studied, beyond reports of aberrant velocities in mutants, such as *inv* [23]. As flow is only required on the left side of the LRO [43], and a few cilia are sufficient to break symmetry [44], the vigor of flow may not be of too much relevance. However, the case is open; studies that assess physical flow parameters are few, and it may be profitable to re-investigate mutants that otherwise are hard to interpret. Absence and immobility of cilia have, however, been analyzed. Both should result in the absence of Nodal cascade induction, which is, however, not observed. Immotile cilia, such as in *iv* mutant embryos, cause randomized *nodal1* expression, which is counterintuitive, while mutants that lack cilia, such as *kif3a* [45] and *ift88* [46,47,48], display bilateral expression, which asks for a mechanistic explanation as well (and we shall come back to both scenarios below).

### 2.3. Flow Cargo

The question whether or not there is cargo that flow transports to the left has been controversially discussed in the field for more than a decade. Early evidence for the presence of so-called Nodal vesicular parcels, or NVPs [49,50], have not been followed up until recently, when the prevailing view that flow itself triggers the flow sensor [51,52] it has been challenged [53,54], but published novel evidence on this issue is still missing. For our hypothetical discussion, the presence, nature and mode of action of NVPs are irrelevant, though. If it were and cargo would be traveling along with leftward flow, this cargo could be present or absent, and it could reach the flow sensors in the presence or absence of leftward flow, for example by diffusion. This is when cases of randomized induction of the Nodal cascade become interesting. In the *iv* mutant, for example, cargo could be released at the LRO and reach the flow sensors on the left and right margin of the LRO in a stochastic manner. This scenario is still not totally satisfying, as the distribution of cases (25% each) asks for a mechanistic explanation. Taking some sort of cargo into account, however, helps the discussion. But, why should absence of cilia, such as in the *kif3a* or *ift88* mutants, be different and result in bilateral induction? In that case, one would need to argue that cargo release and the presence of cilia are coupled. There are several possible scenarios, such as, for example, cargo release from the tip of cilia (in form of exosomes). Care should be applied, however, as cilia mutants impact on very many signaling pathways, some of which may impact on LR patterning downstream of Nodal. Anyways, the cargo option in our opinion should be considered in cases of randomized Nodal expression patterns in the LPM, and the nearer future should tell whether NVPs exist beyond mouse, and when and how they act.

### 2.4. Flow Sensor

The flow sensor should be intimately linked to flow and cargo. In the absence of its trigger, the sensor should not be activated, i.e., Nodal should not be induced. In order to assure robust induction of the Nodal cascade on the left and not the right side of the LPM, the trigger must be locked in the absence of flow and/or cargo. The sensor could be present or absent, and it could be activated on the left, the right, on both or neither side. There should be an absolute correlation between sensor status (on/off) and Nodal cascade activation in the LPM at a slightly later stage. Conceptually, the sensor is the simplest player in the game, as it is this main gateway that should decide on laterality specification [15]. Experimental manipulations of the sensor should overrule all of the upstream events and should be autonomously capable of inducing the cascade on the side where it is touched off. Central to the sensor function is Nodal and its inhibitor Dand5 (formerly known as Coco in frog, charon in zebrafish, or cerl2 in mouse); both are co-expressed in the sensory cells at the LRO margin [55,56,57,58,59,60], or in neighboring cells in the case of zebrafish, where the transformation of the flat epithelium of primitive fish LRO, such as in the sturgeon [30], into the sphere of Kupffer’s vesicle (KV) probably placed these cells just next to the KV. Experimental manipulations of both factors in these very cells underscore their central role: Nodal is strictly required on the left side of the LRO. Its presence in the sensor on the right is not required for left-sided LPM activation. Dand5, in contrast, has to be repressed on the left in order to de-repress Nodal action. When Dand5 disappears from the right sensor and Nodal is present, the cascade gets activated on both sides. Nodal and Dand5 are able to overrule a loss of the flow generator: flow-independent repression of Dand5 activates Nodal, as does the overexpression of Nodal itself. Dand5 depends on Nodal as the left determinant, as in the absence of Nodal in the sensor, Dand5 manipulations remain without consequences. Artificial flow can trigger the sensor in the absence of cilia or ciliary motility [51,61], demonstrating that flow is the natural activator of the sensor, but whether or not this artificial flow carries along cargo remains to be seen. Sensor manipulations are extremely efficient, at least in the frog *Xenopus*, where manipulations can be performed in a sided manner, and in the mouse, reaching efficiencies of close to one hundred percent [58], numbers that are never encountered with any other experimental or genetic manipulation. How flow sets off the sensor, i.e., represses Dand5, is a matter of intense research. Genes that fulfill characteristics, such as Nodal and Dand5, qualify for this process, i.e., flow sensing: they should be highly efficient in knockout or knockdown situations and they should act in a strictly sided manner. Screening the literature for factors complying with these criteria might uncover genes that are involved in sensor function; *pkd2* and *pkd1l1* are certainly good examples [62,63].

### 2.5. Signal Transfer

Finally, the signal that is generated in the sensor needs to transfer from the LRO to the LPM. The transfer system must be present on either side, otherwise bilateral induction of the cascade would not be possible. In its absence, Nodal should never get activated in the LPM. However, contrary to the sensor, it strictly depends on the upstream chain of events and cannot be activated in the absence of the signal. The conceptually most parsimonious scenario, namely that Nodal itself gets transferred, is fully compatible with the available experimental data. While *gdf1/gdf3*, which is expressed in the sensor and complex with Nodal, is required for de-repressed Nodal to transfer to the LPM [60,64], more specific transfer mutants are not known, indicating that the mechanism relies on available cell biological characteristics such as the extracellular matrix or coupling of cells by gap junctions [65,66,67]. Mutations in any such functions would likely impact on other processes as well, obscuring an LR-specific function. Contrary to all the other functions that are discussed above, transfer is likely permissive in nature and not instructive, which is why Nodal cascade expression patterns in our opinion do not reveal a lot in that context.

### 2.6. Precautions

Besides the above-mentioned possibility of full or partial compensation of germline mutations [21], which might blur the function of a given gene when judged by the resulting LPM marker gene expression patterns, genes might act in more than one function, such as Nodal itself, which is required to set up the flow generator [73], in the sensor [74] and in the LPM [75,76]. Some ciliary genes certainly are required in the flow generator and the sensor as well, which has to be taken into account when analyzing the Nodal cascade. Despite these restrictions, we are convinced that a careful re-evaluation of LPM gene expression patterns might be beneficial to place factors in the progress of symmetry breaking, as does the assessment of novel factors that keep to be identified. Such analyses need of course be restricted to model organisms, in which a ciliated LRO has been demonstrated. In vertebrates, this excludes birds and reptiles, which likely have lost these mechanisms and have come up with an alternative strategy of left-asymmetric Nodal cascade induction (cf. [14] for a detailed discussion). Such a loss might even have happened in some mammalian groups, as indicated by the absence of a recently described novel LR determinant of unknown function, the matrix metalloproteinase Mmp21, in the cetartiodactyla, a systematic group of mammals that includes whales and even-toed ungulates [77]. That even-toed ungulates differ from other mammals has been previously suspected, as a ciliated LRO is absent in pig embryos [78].

## 3. Conclusions

In the more than 20 years that have passed since the first description of asymmetric gene expression in the left LPM of the chicken embryo [6], a great many of factors have been involved in the process of laterality determination. Despite all the progress, large gaps remain in our understanding of the molecular and cellular mechanisms that drive organ asymmetry. The majority of vertebrates use a cilia-dependent mode of symmetry breaking; in these cases, the assessment of Nodal cascade gene expression patterns in the LPM can tell us much about the underlying defects. We have argued here that randomized patterns are the result of defects in flow generation, while symmetric patterns arise from defective flow sensing. These different LPM patterns often are not discussed, or only very low cases numbers are reported when a new factor is presented in the literature; any deviation from the norm is taken as evidence for a general role in LR development. While this is correct, important information may be lost when the differences in efficacies and patterns are not pinpointed, evaluated, and weighed. The basic conceptual framework is there; we need to fill in the gaps. It should be profitable to discuss the options any result gives us, rather than concealing our uneasiness. The Nodal cascade has started the molecular analysis of LR analysis, and it remains at center stage now that we tackle the nature of flow generator and sensor.

## Figures and Tables

**Table 1:** Nodal cascade gene expression patterns in selected LR mutants.

Process	Gene/Treatment	Species	Mutant	Morphant	Nodal Cascade *	References
**LRO specifier**	*brachyury*	mouse	*T/T*		absent	[34,36]
			*T^Wis/TWis^*		absent	
		fish	*ntl*		absent	[35]
						SK & MB
		frog	*Xbra*	TBMO	absent	(unpublished)
**LRO flow**	methyl cellulose	*Xenopus*			absent	[44,68]
		mouse			bilateral	
**flow generator**	*FOXJ1*	Zebrafish		TBMO	random	[69,70]
		mouse	*Foxj1^neo/neo^*		random	
					absent, bilateral	
	*KIF3A*	mouse	*-/-*		bilateral	[45,61]
	*RFX2*	*Xenopus*		SBMO	absent, bilateral	[71,72]
		zebrafish		TBMO	absent	
**cilia motility**	*DNAH11*	mouse	*Dnah11^iv/iv^*		random	[5,19,43]
	*DNAH9*	*Xenopus*		TBMO	absent	
	*DNAH5*	*Xenopus*		TBMO	absent	
	*DYX1C1*	zebrafish		TBMO	absent	
**ciliapolarity**	*VANGL1*	mouse	*Vangl1^gt/gt^*		bilateral	[40,41]
	*VANGL2*		*Vangl2^−/−^*		bilateral	
		*Xenopus*		TBMO	absent	
**LRO sensor**	*NODAL*	mouse			absent	[55]
		zebrafish		TBMO	random	[56,57,58]
		*Xenopus*		TBMO	absent	
	*DAND5*	mouse	*-/-*		random	[58,59,60]
		*Xenopus*		TBMO	bilateral	
		zebrafish		TBMO	bilateral	
	*GDF1/GDF3*	mouse	*-/-*		absent	[60]
		zebrafish		TBMO	absent	
		zebrafish	*mz-/-*		absent	
	*PKD2*	mouse	*Pkd2^lacZ/lacZ^*		absent	[62]
		zebrafish		TBMO	bilateral	
		zebrafish		SBMO	absent	
	*PKD1L1*	mouse	*Pkd1l1^rks/rks^*		absent	[63]
		medaka	*abc^aA12^*		absent	

* Expression of *nodal*, *pitx2* and *lefty2* in the left lateral plate mesoderm.
